# Lifestyle and health-related quality of life: A cross-sectional study among civil servants in China

**DOI:** 10.1186/1471-2458-12-330

**Published:** 2012-05-04

**Authors:** Jun Xu, Jincai Qiu, Jie Chen, Liai Zou, Liyi Feng, Yan Lu, Qian Wei, Jinhua Zhang

**Affiliations:** 1 Department of Sanitation Economy Administration, Nanfang Hospital, Southern Medical University, Guangzhou, Guangdong Province, PR China; 2 School of Public Health and Tropical Medicine, Southern Medical University, Guangzhou, Guangdong Province, PR China; 3 Medicare office, First People’s Hospital, Shunde, Foshan, Guangdong Province, PR China; 4 School of Nursing, Southern Medical University, Guangzhou, Guangdong Province, PR China

**Keywords:** Health-related quality of life, SF-36, Civil servants, Lifestyle

## Abstract

**Background:**

Health-related quality of life (HRQoL) has been increasingly acknowledged as a valid and appropriate indicator of public health and chronic morbidity. However, limited research was conducted among Chinese civil servants owing to the different lifestyle. The aim of the study was to evaluate the HRQoL among Chinese civil servants and to identify factors might be associated with their HRQoL.

**Methods:**

A cross-sectional study was conducted to investigate HRQoL of 15,000 civil servants in China using stratified random sampling methods. Independent-Samples *t*-Test, one-way ANOVA, and multiple stepwise regression were used to analyse the influencing factors and the HRQoL of the civil servants.

**Results:**

A univariate analysis showed that there were significant differences among physical component summary (PCS), mental component summary (MCS), and TS between lifestyle factors, such as smoking, drinking alcohol, having breakfast, sleep time, physical exercise, work time, operating computers, and sedentariness (P < 0.05). Multiple stepwise regressions showed that there were significant differences among TS between lifestyle factors, such as breakfast, sleep time, physical exercise, operating computers, sedentariness, work time, and drinking (P < 0.05).

**Conclusion:**

In this study, using Short Form 36 items (SF-36), we assessed the association of HRQoL with lifestyle factors, including smoking, drinking alcohol, having breakfast, sleep time, physical exercise, work time, operating computers, and sedentariness in China. The performance of the questionnaire in the large-scale survey is satisfactory and provides a large picture of the HRQoL status in Chinese civil servants. Our results indicate that lifestyle factors such as smoking, drinking alcohol, having breakfast, sleep time, physical exercise, work time, operating computers, and sedentariness affect the HRQoL of civil servants in China.

## Background

The World Health Organization (WHO) defines quality of life as the living conditions associated with the corresponding goals, expectations, standards, and concerns of each individual living in different cultural systems [1-3]. The quality of life, as a new health indicator, is not only concerned about how long patients can survive, but more concerned about how well patients live. The first measurement of the quality of life is the United States national measurement report in 1960 by the United States Dwight D. Eisenhower National Goal Committee [4]. After the 1980s, the assessment of the quality of life in the allocation of health resources, the choice of patient (individual) treatments, the comparison of various treatments in clinical trials, and quality of life measurement in healthy people has been widely applied, and the WHO proposes the concept of health-related quality of life (HRQoL) [5]. The quality of life is mainly measured using rating scales, such as the Short Form 36 items (SF-36) scale [6]. The SF-36 scale has demonstrated reliability and validity in assessing the health of the general population, is a well-recognised outcome measure for health status, and has been used extensively as an important health outcome indicator [7]. With respect to the effects of factors on health, findings from previous studies among civil servants have shown how HRQoL is associated with disease, psychological problems, and so on [8-11]. However, very few studies examined whether and how HRQoL is associated with lifestyle and health among civil servants. This study is unique because the sample was nationally representative, and assessed the association between HRQoL and lifestyle factors, compared to differences between different lifestyle groups. Therefore, in this study we aimed to assess self-reported HRQoL among Chinese civil servants using the SF-36 instrument; the association between lifestyle factors and HRQoL, and the impact of lifestyle factors, such as smoking, drinking alcohol, having breakfast, sleep time, physical exercise, work time, operating computers, and sedentariness on HRQoL. Studying this issue can provide important information for local health care policy makers and researchers to consider at which levels effective public health interventions should be implemented to improve the HRQoL of civil servants and to further confirm the universal nature of association between lifestyle factors and health across cultural boundaries.

## Methods

### Study population and data collection

This was a cross-sectional survey of a random sample of civil servants selected from five regions (North China, South China, Central China, Northwest China, and Northeast China) in China. Civil servants in the present study refer to those who perform public duties and have been included in the state administrative system with wages and welfare provided by the state public finance [12]. The sampling method was based on a stratified random sampling approach. These five regions represent typical levels in respect of the regions scale and geographical distribution. North China, South China, and Central China are typical of greatly developed regions. Northeast China is representative of moderately developed regions. Northwest China is representative of underdeveloped regions. Overall, these five regions represent the characteristics of different types. Therefore, the survey of civil servants from these regions could well represent the HRQoL status of civil servants in China. Affiliation such as provincial, municipal, county, town, and village were randomly selected in five regions. All the participants had to be aged 18 years or older, including retired ones. Age was categorised into groups: 18–24 years, 25–34 years, 35–44 years, 45–54 years, and 55 years or above. Proportionate allocation sampling was used to identify a sampling fraction for each district, age segment, and gender (male/female = 2/1). The sample sizes of each region, which can be calculated by formula [13], were 2,401. Taking the loss rate into account, we expand the sample size by adding 10%. Considering the smallest sample size and different regions have different proportion of civil servants, the sample size of the civil servants living in North China, South China, Central China, Northwest China, and Northeast China were 4,050; 2,760; 2,580; 3,030; 2,580, respectively. A total of 15,000 civil servants in China were recruited in this study, and 93.47% (14,021) responded to the survey.

All the respondents gave written informed consent to all assessments reported and the study was approved by the Nanfang hospital ethics committee (see Additional file 1).

### Measurement tools

A questionnaire survey was conducted. The questionnaire (see Additional file 2) involved two parts. The first part included general situations of Chinese civil servants. The second part was the HRQoL measurement scale: the SF-36.

General situations included socio-demographic factors, including gender, age, ethnic, height, weight, region, marital status, education, monthly income, and living place. Lifestyle included smoking, drinking alcohol, having breakfast, sleep time, physical exercise, work time, sedentariness, and life experience.

SF-36 is a form containing 36 items divided into eight dimensions of health using a multi-item scale [14]: physical functioning (PF), role-physical (RP), bodily pain (BP), general health (GH), vitality (VT), social functioning (SF), role-emotional (RE), and mental health (MH). The eight scales were scored from 0 to 100 (worst to best possible health status). The SF-36 is a reliable and validated instrument [15,16]. The physical health-related dimensions (physiological function, role-physical, bodily pain, general health) and mental health-related dimensions (vitality, social functions, role emotional, mental health) in SF-36 are assigned to physical component summary (PCS) and mental component summary (MCS) [17,18]. SF-36 was scored on the basis of national uniform standards in China. The score was then re-calculated across the dimensions:

(1) $$transformed\;score=\frac{Actual\;raw\;score-lowest\;possible\;raw\;score}{possible\;raw\;score\;range}\times 100$$

The raw score of each dimension was derived by summing the item scores, and then converted to a value ranging from 0 (worst possible health status measured by the questionnaire) to 100 (best possible health status) [19].

### Quality control

The investigator explained to subjects how to fill in the scale before the test. Then, subjects fill the questionnaire independently on the basis of their understanding of each item. Afterwards, we rejected questionnaires that were less than 80% complete, had low writing quality (for instance, all of the questions were with the same answer), and those questionnaires that had identical answers. The database was established by Epidata3.02, and double input was conducted to ensure the lowest deviation.

### Statistical analysis

Epdata3.02 (Military Medical Science Press,Beijing, China) was adopted to manage the data in the survey. Statistical analysis was carried out using SPSS15.0 for Windows, version 15.0 (SPSS, Inc., Chicago, Illinois). The statistical description of the clinical and socio-demographic variables was denoted by frequencies, percentages, means, and standard deviations. After calculating descriptive statistics, t-tests and a one-way ANOVA were used to assess group differences with respect to their statistical significance. Additionally, multivariate stepwise regression analysis was performed to assess the impact of gender, educational level, and living arrangements on HRQoL.

## Results

### Demographic characteristics of the participants

Totally, 15,000 participants received the interview and 14,021 (93.47%) responded to the survey. Among them, 12,941 (92.30%) were included in the data analyses. As shown in Table 1, 7,953 (61.46%) were males and 4,904 (37.90%) were females. The percentages of the civil servants in North China, South China, Central China, Northwest China, and Northeast China were 3,610 (27.90%); 2,492 (19.26%); 1,973 (15.25%); 2,695 (20.83%); 2,171 (16.78%), respectively. The means (SD) age of the participants was 38.72 (9.43).

**Table 1:** Frequency distribution of civil servants’ demographical and lifestyle characteristics (N = 12941)

**Factors**	**Groups**	**N**	**%**	**Factors**	**Groups**	**N**	**%**
**Gender**	**Male**	7953	61.46	**Breakfast**	**Never**	249	1.92
	**Female**	4904	37.90		**Sometimes**	1386	10.71
	**Missing value**	84	0.65		**Often**	4355	33.65
**Age**	**18~**	459	3.55		**Every day**	6860	53.01
	**25~**	4209	32.52		**Missing value**	91	0.70
	**35~**	4377	33.82	**Sleeping time**	**Below 4**	110	0.85
	**45~**	3094	23.91		**4~**	448	3.46
	**55~**	692	5.35		**6~**	6251	48.30
	**Missing value**	110	0.85		**8~**	5925	45.78
**Region**	**North China**	3610	27.90		**10~**	132	1.02
	**South China,**	2492	19.26		**Missing value**	75	0.58
	**Central China**	1973	15.25	**Physical exercise**^**a**^	**Little or not**	4683	36.19
	**Northwest China**	2695	20.83		**1 ~ 4**	1939	14.98
	**Northeast China**	2171	16.78		**5 ~ 8**	3793	29.31
**Marital status**	**Non-married**	1845	14.26		**9 ~ 12**	1050	8.11
	**Married**	10619	82.06		**Above 12**	1345	10.39
	**Divorced/Widowed/Other**	331	2.56		**Missing value**	131	1.01
	**Missing value**	146	1.13	**Work time**	**Below 6**	1425	11.01
**Education**	**Middle school**	105	0.81		**6~**	9506	73.46
	**High school**	685	5.29		**10~**	1273	9.84
	**College**	11183	86.42		**12~**	386	2.98
	**Under graduate**	738	5.70		**Missing value**	351	2.71
	**Missing value**	230	1.78	**Sedentariness**^**b**^	**Rarely**	5293	40.90
**Smoke**	**No**	9082	70.18		**Sometimes**	2814	21.74
	**Yes**	3807	29.42		**Often**	2841	21.95
	**Missing value**	52	0.40		**Always**	1913	14.78
**Drink**	**Never**	2990	23.10		**Missing value**	80	0.62
	**A little**	7034	54.35				
	**A lot**	2782	21.50				
	**Missing value**	135	1.04				

**a:** Little or not, 1 ~ 4 times a month, 5 ~ 8 times a month, 9 ~ 12 times a month, Above 12 times a month.

**b:** Operating computer and sedentariness.

### HRQoL of Chinese civil servants

Table 2 shows the scores of HRQoL among Chinese civil servants.

**Table 2:** HRQoL of Chinese civil servants (N = 12941)

**Scale**		**Means**	**SD**
**Physical health scales(PCS)**		76.05	14.71
	**Physical Functioning (PF)**	88.50	15.58
	**Role Physical (RP)**	79.24	32.64
	**Bodily Pain (BP)**	75.95	19.56
	**Mental Health (MH)**	63.00	19.51
**Mental health scales(MCS)**		72.01	15.89
	**Role Emotional (RE)**	68.83	17.39
	**Social Functioning (SF)**	78.62	19.36
	**Vitality (VT)**	74.39	37.29
	**General Health (GH)**	72.15	17.10
**Total Scores (TS)**		73.99	14.15

### Univariate analysis

Table 3 and 4 shows the relationships between the lifestyle factors and HRQoL.

**Table 3:** HRQoL associated with lifestyle factors in physiological among Chinese civil servants (N = 12941)

**Factors**	**Groups**	**Physical health scales (PCS)**				
		**PF**	**RP**	**BP**	**GH**	**PCS summary measure**
**Smoke**	**No**	89.04 ± 14.86	80.44 ± 32.03	76.03 ± 19.32	63.43 ± 19.34	50.83 ± 8.24
	**Yes**	87.23 ± 17.10	76.47 ± 33.82	75.67 ± 20.11	61.97 ± 19.87	50.00 ± 9.00
	**t**	5.698	6.173	0.930	3.868	4.906
	**P**	0.000	0.000	0.352	0.000	0.000
**Drink**	**Never**	86.54 ± 17.93	79.16 ± 32.37	75.34 ± 20.95	63.05 ± 19.63	49.95 ± 9.17
	**A little**	89.41 ± 14.35	79.96 ± 32.22	76.12 ± 18.91	63.16 ± 19.57	50.87 ± 8.09
	**A lot**	88.68 ± 15.45	78.29 ± 33.46	76.30 ± 19.65	62.97 ± 19.05	50.76 ± 8.59
	**F**	35.901	2.733	2.145	0.102	12.723
	**P**	0.000	0.065	0.117	0.903	0.000
**Breakfast**	**Never**	73.72 ± 25.60	65.61 ± 35.75	62.23 ± 25.23	56.65 ± 17.10	44.49 ± 11.13
	**Sometimes**	83.30 ± 20.17	70.39 ± 36.07	70.21 ± 20.68	58.19 ± 19.41	48.00 ± 10.00
	**Often**	88.20 ± 15.73	77.72 ± 33.12	75.77 ± 19.14	61.82 ± 18.89	50.47 ± 8.36
	**Every day**	90.36 ± 13.12	82.60 ± 30.84	77.75 ± 18.94	65.00 ± 19.71	51.44 ± 7.89
	**F**	166.341	77.321	102.348	67.226	112.097
	**P**	0.000	0.000	0.000	0.000	0.000
**Sleeping time**	**Below 4**	74.08 ± 28.35	66.45 ± 34.58	62.37 ± 25.75	54.99 ± 19.51	44.30 ± 12.22
	**4~**	74.39 ± 25.72	65.03 ± 36.20	62.47 ± 21.23	53.04 ± 19.16	43.75 ± 11.35
	**6~**	87.99 ± 15.26	77.69 ± 33.44	74.30 ± 19.56	60.98 ± 19.63	50.05 ± 8.59
	**8~**	90.52 ± 13.50	82.31 ± 30.93	78.96 ± 18.46	65.99 ± 18.81	51.83 ± 7.57
	**10~**	83.83 ± 20.41	74.85 ± 35.19	76.60 ± 21.43	64.43 ± 20.91	49.20 ± 10.58
	**F**	152.062	43.233	116.845	87.831	131.978
	**P**	0.000	0.000	0.000	0.000	0.000
**Physical exercise**	**Little or not**	87.56 ± 15.70	78.15 ± 34.09	74.67 ± 19.82	59.90 ± 20.05	49.79 ± 8.58
	**1 ~ 4**	87.32 ± 16.86	76.55 ± 33.69	74.51 ± 19.96	63.89 ± 18.53	50.15 ± 8.74
	**5 ~ 8**	88.94 ± 15.70	79.51 ± 31.61	76.37 ± 18.62	63.15 ± 18.74	50.86 ± 8.25
	**9 ~ 12**	90.58 ± 14.08	81.18 ± 31.02	78.37 ± 20.61	67.04 ± 19.00	52.22 ± 8.55
	**Above 12**	91.08 ± 12.91	84.67 ± 29.26	79.64 ± 18.96	69.10 ± 19.59	52.13 ± 7.94
	**F**	21.996	14.945	24.271	76.231	33.571
	**P**	0.000	0.000	0.000	0.000	0.000
**Work time**	**Below 6**	86.70 ± 17.38	79.26 ± 32.63	73.87 ± 20.23	62.42 ± 19.32	49.60 ± 9.17
	**6~**	89.07 ± 14.76	80.64 ± 31.62	76.77 ± 18.98	63.95 ± 19.15	50.92 ± 8.08
	**10~**	87.55 ± 16.78	70.66 ± 36.86	72.84 ± 20.57	58.41 ± 20.70	49.49 ± 9.04
	**12~**	85.98 ± 19.70	73.05 ± 36.67	72.99 ± 24.17	59.76 ± 23.09	50.35 ± 11.67
	**F**	15.757	40.167	24.808	35.051	18.675
	**P**	0.000	0.000	0.000	0.000	0.000
**Sedentariness**	**Rarely**	90.09 ± 13.30	80.90 ± 31.75	77.12 ± 18.26	64.07 ± 19.18	51.19 ± 7.70
	**Sometimes**	88.11 ± 15.84	79.07 ± 32.57	77.69 ± 19.66	64.33 ± 18.74	50.82 ± 8.41
	**Often**	90.03 ± 13.36	79.89 ± 32.96	74.22 ± 19.90	60.12 ± 20.68	50.66 ± 8.49
	**Always**	82.70 ± 21.41	74.55 ± 33.91	72.83 ± 21.76	62.59 ± 19.36	48.63 ± 10.18
	**F**	120.206	18.195	37.654	30.707	44.334
	**P**	0.000	0.000	0.000	0.000	0.000

Statistical methods: Independent-Samples *t* Test or One-Way ANOVA.

**Table 4:** HRQoL associated with lifestyle factors in psychological among civil servants (N = 12941)

**Factors**	**Groups**	**Mental health scales (MCS)**				
		**VT**	**SF**	**RE**	**MH**	**MCS summary measure**
**Smoke**	**No**	69.31 ± 17.22	79.30 ± 19.01	75.10 ± 37.00	72.69 ± 16.80	50.86 ± 9.31
	**Yes**	67.64 ± 17.74	77.00 ± 20.07	72.67 ± 37.92	70.86 ± 17.74	49.98 ± 9.67
	**t**	4.910	6.022	3.342	5.431	4.816
	**P**	0.000	0.000	0.001	0.000	0.000
**Drink**	**Never**	68.62 ± 17.64	77.38 ± 20.56	76.58 ± 35.52	71.20 ± 17.72	50.72 ± 9.21
	**A little**	69.02 ± 17.33	79.62 ± 18.80	75.07 ± 36.72	72.66 ± 16.75	50.82 ± 9.40
	**A lot**	68.83 ± 17.20	77.86 ± 19.14	71.05 ± 39.67	72.17 ± 17.18	50.12 ± 9.62
	**F**	0.591	17.712	17.570	7.694	5.700
	**P**	0.554	0.000	0.000	0.000	0.003
**Breakfast**	**Never**	58.90 ± 17.20	62.34 ± 23.04	61.74 ± 39.12	56.62 ± 16.81	44.64 ± 9.29
	**Sometimes**	62.52 ± 17.75	70.68 ± 21.90	64.52 ± 39.45	64.91 ± 17.30	46.91 ± 9.37
	**Often**	67.54 ± 17.44	77.02 ± 19.11	72.10 ± 38.17	70.55 ± 17.02	49.61 ± 9.36
	**Every day**	71.29 ± 16.75	81.9017.84	78.34 ± 35.54	75.25 ± 16.20	52.21 ± 9.10
	**F**	146.219	223.547	74.366	253.684	195.408
	**P**	0.000	0.000	0.000	0.000	0.000
**Sleeping time**	**Below 4**	60.85 ± 16.70	63.08 ± 24.15	58.64 ± 38.82	59.56 ± 18.22	45.41 ± 8.87
	**4~**	58.37 ± 19.21	62.44 ± 23.97	62.35 ± 38.75	59.74 ± 18.14	45.47 ± 9.61
	**6~**	67.06 ± 17.45	77.71 ± 19.46	71.32 ± 38.80	70.87 ± 17.15	49.76 ± 9.67
	**8~**	71.57 ± 16.59	81.10 ± 17.88	78.92 ± 34.79	74.68 ± 16.23	51.96 ± 8.87
	**10~**	69.53 ± 17.74	80.14 ± 19.46	72.59 ± 38.65	72.06 ± 18.32	51.51 ± 9.34
	**F**	102.465	128.853	49.876	119.371	87.382
	**P**	0.000	0.000	0.000	0.000	0.000
**Physical exercise**	**Little or not**	66.31 ± 18.65	77.99 ± 19.80	73.25 ± 38.46	70.84 ± 17.68	49.95 ± 9.91
	**1 ~ 4**	68.59 ± 16.46	76.18 ± 19.49	72.13 ± 37.32	70.73 ± 17.31	49.92 ± 9.07
	**5 ~ 8**	69.15 ± 16.26	78.78 ± 18.79	74.51 ± 36.65	71.89 ± 16.38	50.51 ± 9.04
	**9 ~ 12**	71.42 ± 15.65	78.53 ± 19.45	73.28 ± 38.12	73.46 ± 16.56	50.65 ± 9.11
	**Above 12**	75.00 ± 16.60	84.14 ± 17.90	81.73 ± 33.48	78.71 ± 15.38	54.08 ± 8.70
	**F**	74.817	36.820	16.184	62.813	55.101
	**P**	0.000	0.000	0.000	0.000	0.000
**Work time**	**Below 6**	69.47 ± 16.48	78.38 ± 19.93	73.83 ± 36.55	73.00 ± 16.48	51.22 ± 8.99
	**6~**	69.92 ± 16.74	79.47 ± 18.74	76.78 ± 35.73	73.11 ± 16.62	51.23 ± 9.11
	**10~**	62.35 ± 20.10	74.01 ± 21.61	63.96 ± 42.45	66.51 ± 19.18	46.95 ± 10.75
	**12~**	60.64 ± 19.93	72.39 ± 21.66	48.79 ± 45.08	65.68 ± 18.65	44.66 ± 10.08
	**F**	103.041	43.833	109.333	77.222	134.325
	**P**	0.000	0.000	0.000	0.000	0.000
**Sedentariness**	**Rarely**	70.09 ± 16.76	80.76 ± 17.70	76.31 ± 36.73	74.17 ± 16.43	51.50 ± 9.22
	**Sometimes**	70.49 ± 16.68	78.41 ± 19.67	75.98 ± 35.77	72.50 ± 16.92	51.05 ± 9.10
	**Often**	66.68 ± 18.64	79.27 ± 19.15	71.30 ± 39.54	71.33 ± 17.01	49.67 ± 10.03
	**Always**	66.26 ± 17.51	72.26 ± 22.05	71.73 ± 36.86	67.44 ± 18.21	48.95 ± 9.16
	**F**	46.897	93.495	16.207	76.918	47.384
	**P**	0.000	0.000	0.000	0.000	0.000

Statistical methods: Independent-Samples *t* Test or One-Way ANOVA.

Significant differences among PCS, MCS, and TS between lifestyle factors were found (P < 0.05). The smoking group had lower scores in PCS, MCS, and TS than the non-smoking group; civil servants who drank a little had higher scores in PCS, MCS, and TS than civil servants who never drank or drank a lot. The scores of the servants who never ate breakfast were significantly lower than those of other groups. The group in which civil servants had 8–10 hours sleep had higher scores in PCS, MCS, and TS than other groups. The group in which civil servants had 4 hours or less sleep had the lowest scores. Civil servants who participated in physical exercise more than 12 times a month had higher scores in PCS, MCS, and TS. Civil servants who had little or no physical exercise had the lowest scores. Civil servants who worked less than 10 hours a day had higher scores in PCS, MCS, and TS than those who worked more than 10 hours a day. Civil servants always operating computers and who were mostly sedentary had lower scores in PCS, MCS, and TS than those in other groups.

### Multiple stepwise linear regressions analysis

The SF-36 scale total score was used as the dependent variable, and lifestyle factors, as independent variables. Multiple linear regression was conducted to analyse the social-demographic factors that affect Chinese civil servants’ HRQoL. The regression results showed that there were significant differences among PCS, MCS, and TS between lifestyle factors, such as sleep time, having breakfast, physical exercise, operating computers, sedentariness, drinking alcohol, and smoking in PCS; having breakfast, sleep time, physical exercise, operating computers, sedentariness, and work time in MCS; having breakfast, sleep time, physical exercise, operating computers, sedentariness, work time, and drinking alcohol in TS (P < 0.05) (see Table 5).

**Table 5:** Relationships between the lifestyle factors and HRQoL (Multivariate analysis N = 12941)

**Scales**	**B**	**SE**	**β**	**t**	**P**	**95%*CI***	
**PCS**							
** Sleeping time**	3.743	0.210	0.159	17.830	0.000	3.332	4.155
** Breakfast**	2.491	0.177	0.127	14.041	0.000	2.143	2.839
** Physical exercise**	1.200	0.097	0.109	12.416	0.000	1.011	1.390
** Operating computer and sedentariness**	−0.765	0.083	−0.080	−9.186	0.000	−0.929	−0.602
** Drink**	0.691	0.208	0.032	3.314	0.001	0.282	1.099
** Smoke**	−0.682	0.309	−0.021	−2.209	0.027	−1.287	−0.077
**MCS**							
** Breakfast**	3.842	0.188	0.181	20.428	0.000	3.473	4.211
** Sleeping time**	3.565	0.224	0.140	15.934	0.000	3.126	4.003
** Physical exercise**	1.271	0.103	0.107	12.335	0.000	1.069	1.473
** Operating computer and sedentariness**	−1.046	0.089	−0.101	−11.788	0.000	−1.220	−0.872
** Work time**	−1.628	0.166	−0.084	−9.792	0.000	−1.954	−1.302
**TS**							
** Breakfast**	3.195	0.167	0.169	19.125	0.000	2.868	3.523
** Sleeping time**	3.675	0.199	0.162	18.457	0.000	3.285	4.066
** Physical exercise**	1.228	0.092	0.116	13.392	0.000	1.048	1.408
** Operating computer and sedentariness**	−0.909	0.079	−0.099	−11.522	0.000	−1.064	−0.755
** Work time**	−0.954	0.148	−0.055	−6.440	0.000	−1.245	−0.664
** Drink**	0.426	0.181	0.020	2.348	0.019	0.070	0.782

Statistical methods: multiple stepwise linear regression analysis.

## Discussion

This study has shown that Chinese civil servants who are frequently under excessive pressure of work, interpersonal relationships, and family stress may have physical and mental disorders, serious psychological fatigue, and psychological problems. The physical and mental disorders may in turn increase the pressure and lead to sub-health and various diseases [20,21], and may eventually reduce the efficiency of work and decrease HRQoL.

Personal habits will have an impact on the HRQoL of Chinese civil servants. This research shows that lifestyle factors such as smoking, drinking alcohol, having breakfast, sleep time, physical exercise, work time, operating computers, and sedentariness have a strong association with civil servants’ HRQoL.

Bad living habits such as smoking and drinking are major factors causing the decline of physical condition [22,23]. Previous studies have shown that under the influence of alcohol, drinking a little can make blood vessels expand and relax. Alcohol goes into the blood circulation and has a certain inhibition on the sympathetic system. Therefore, moderate drinking may protect the cardiovascular and cerebrovascular system [24,25]. As the result showed, civil servants who drank a little had higher HRQoL than civil servants who never drank or drank a lot. That means moderate drinking has benefits on civil servants’ quality of life. In this study, the total drinking rate among civil servants is 62.52%. There may be two reasons; first, they participate in some social drinking or alcoholism because of friendly gatherings, work entertainment, personal appetite, and many other reasons. Second, in order to pursue self-health, they have a small amount of alcohol safely at home under the influence of the concept that moderate alcohol consumption has a protective effect on the cardiovascular system. For civil servants, risks of social drinking or alcoholism are enormous. A good grasp of the safety of self-protective drinking is difficult, and a little excessive drinking not only has no benefits but is extremely harmful to health. Therefore, government departments should pay enough attention to this problem. In the study, the total smoking rate among civil servants is 34.27%. Smoking is a great threat to human life, and induces death by cardiovascular disease of 11% of the total population in the world [22]. A Norwegian study shows that smokers who smoke 1–4 cigarettes a day have more risk of ischemic heart disease than non-smokers [26]. At the same time, the dangers of passive smoking on the human body cannot be ignored. Global researches on passive smoking and lung cancer indicate that there is a causal association between the occurrence of lung cancer and passive smoking [27]. Therefore, smoking can physically cause harm to civil servants. The more smoking, the greater damage there is to civil servants’ physical health and lower PCS scores.

Decline in HRQoL is closely related to poor eating habits and irregular lifestyle. For civil servants, poor diet includes too simple a breakfast or having no breakfast. The body in a state of hunger may easily lead to palpitation, dizziness, mental deficiencies, and so on. In the study, some civil servants had no breakfast or rarely did so, and the proportion of those having breakfast daily was 61.68%. We may see that the phenomenon of irregular diet and no breakfast among civil servants is serious. As a basic human need, food intake affects people’s HRQoL [28]. Scientific diet is the healthy material guarantee. Civil servants should change bad eating habits, have food on time, and pay attention to a reasonable nutrition.

Sleep time is closely related to HRQoL [29]. As a physiological activity, sleep is more important than eating, and all the body’s vital functions are slowed down, completely at the state of rest, recovering and accumulating energy. If one has prolonged insomnia, this will lead inevitably to the irreparability of energy and spirit, and life exhaustion. A British and American study has shown that sleeping less than 6 hours per day is linked to the occurrence of some diseases, and people sleeping more than 8 hours per day show only minor illness with epiphenomenon [23]. As an important physiological process, sleeping not only maintains normal function but also protects the normal metabolism and functions of various organs of body. If one cannot get adequate sleep, many negative manifestations such as sleep deprivation, physical fatigue discomfort, and other negative emotions with tension and anxiety may occur, therefore, leading to a decline in reactions, enthusiasm, abilities in solving problems, and HRQoL. As the previous studies showed above, sleeping problems may lead to poor sleep quality and illness, which might decline HRQoL. This might explain why civil servants who had 8–10 hours sleep had higher scores in PCS, MCS, and TS. In addition, adequate sleep is quite important, which can reduce and relieve stress for mental and emotional balance. Therefore, civil servants should arrange work and study reasonably to ensure adequate sleep.

Physical exercise can reduce the risk of many diseases, such as ischemic heart disease [30], stroke [31], diabetes [32], cognitive disorder [12], and total mortality [33]. And moderate exercise can promote physical and mental health of individuals. Physical exercise can not only improve adaptability of cardiopneumatic and disease resistance, reduce diseases, enhance the body, and avoid obesity, but it can also help maintain a strong energy to improve efficiency at work. Appropriate exercise is conducive to physical and mental health, which can make people relax, eliminate tension, and enhance the immunity and adaptability to the environment. Taking part in various sports activities can improve HRQoL, by exercising, improving community participation, and so on. The study found that 32.58% of civil servants have scarcely done physical exercise. Heavy tasks, personal stress, body fatigue after work, having no interest in sports, and lack of sports site, equipment, and facilities may be the reasons that affect civil servants’ initiative and level of participating in exercise.

This study indicated that work time is associated with HRQoL. Long-term working and work entertainment increase civil servants’ workload directly, reduce their leisure time, and potentially add to their work pressure, which may gradually make satisfaction and enthusiasm for their job weaken with lack of energy. What is more, they result in boredom, anxiety, depression, and other psychological symptoms, and affect individual health and induce the decline of life quality. A five-year follow-up survey of UK White House showed that the danger of civil servants working more than 55 hours per week and suffering from depression or anxiety symptoms is 1.66 times and 1.74 times respectively than civil servants working 35–40 hours per week, and long-term working is excessively dangerous for women civil servants[34]. Many factors such as a long period of overtime, excessive work, and mental stress also affect the health of employees indirectly [35,36]. Therefore, civil servants who worked less than 10 hours a day had higher scores in PCS, MCS, and TS than those who worked more than 10 hours a day.

Operating computers and sedentariness have an adverse effect on health [37]. Civil servants’ characteristics were ‘entertainment-style’, ‘stay-up-late-style’, ‘sitting silently type’, ‘meeting-style’, and ‘thinking-style’. Among these characteristics, ‘stay-up-late–style’, ‘sitting silently type’, ‘meeting-style’, and ‘thinking-style’ usually need long-term operating of computers, which induces a variety of chronic diseases, such as cervical spondylosis, scapulohumeral periarthritis, obesity, fatty liver, hypertension, and hyperlipoidemia. And these diseases have a negative impact on the health of civil servants, followed by the deterioration of health quality and the gradual descent of work efficiency.

### Limitation

In this study, the performance of the questionnaire in a large-scale survey is satisfactory and provides a large picture of the HRQoL of civil servants in China. However, several limitations need to be taken into account when interpreting our findings. Firstly, our use of data from five regions (North China, South China, Central China, Northwest China, and Northeast China) in China limited the applicability of our result to other countries. Secondly, the study design was cross-sectional and it is hence difficult to establish a cause-effect relationship between HRQoL and lifestyle factors. A longitudinal study is needed to investigate the relationship in a future study. Despite these limitations, the results of this analysis provide a large picture of the HRQoL of civil servants in China, which may facilitate further investigation by using a prospective study design.

## Conclusions

In this study, using SF-36, we assessed the association of HRQoL with lifestyle factors, including smoking, drinking alcohol, having breakfast, sleep time, physical exercise, work time, operating computers, and sedentariness in China. The performance of the questionnaire in the large-scale survey is satisfactory and provides a large picture of HRQoL status in Chinese civil servants. The study showed that lifestyle factors such as smoking, drinking alcohol, having breakfast, sleep time, physical exercise, work time, operating computers, and sedentariness affect the HRQoL of Chinese civil servants. Multiple stepwise regressions showed that lifestyle factors such as smoking, drinking, breakfast, sleep time, physical exercise, work time, operating computers, and sedentariness affect the HRQoL of Chinese civil servants.

## Abbreviations

HRQoL = Health-related quality of life; SF-36 = Short form 36 items; One-Way ANOVA = One-Way Analysis of Variance; PCS = Physical Component Summary of the SF-36; MCS = Mental Component Summary of the SF-36; TS = Total Scores of the SF-36; PF = Physical Functioning; RP = Role-Physical; BP = Bodily Pain; GH = General Health; VT = Vitality; SF = Social Functioning; RE = Role-Emotional; MH = Mental Health.

## Competing interests

The authors declare that they have no competing interests.

## Authors’ contributions

JX developed the questionnaire and study design, supervised the analysis and contributed to the final version of the manuscript. JQ who assisted with the survey and data analyses is also the principal authors of this paper. JC, LZ, LF, YL, QW & JZ assisted with the study. All authors have read and approved the final manuscript.

## Funding

This research was supported by the Youth Project of Humanities and Social Sciences,Ministry of Education of China (NO: 10YJCZH192) and the Planned Science and Technology Project of Guangdong Province in China (NO: 2009B080701073).

## Pre-publication history

The pre-publication history for this paper can be accessed here:

http://www.biomedcentral.com/1471-2458/12/330/prepub

## Supplementary Material

Additional file 1 Ethical approval. Click here for file

Additional file 2 Questionnaire. Click here for file
