# Supplementary material for: Lifestyle and health-related quality of life: A cross-sectional study among civil servants in China
Source: BMC Public Health. 2012 May 4;12:330. doi: 10.1186/1471-2458-12-330 (PMC3432623; doi:10.1186/1471-2458-12-330)
Supplement: Additional file 1 — Ethical approval. [file 1471-2458-12-330-S1.doc]

编号：

**公务员与健康相关生存质量状况 调查问卷**

国家教育部人文社会科学研究青年基金项目课题组

广东省科技计划项目课题组

二〇一〇年九月

**现场调查质量控制表(由调查员填写)**

1. 本次调查表由：

□ 被调查者自己完成填写 □ 由他人代替填写

□ 在调查员的协助下，由被调查员自己完成填写

1. 调查表的填写存在缺失项目：□ 无 □ 有, 项
2. 被调查者的依从性问题

□ 配合，主动完成问卷 □ 不配合，在调查员的劝解下完成问卷 □ 不配合，不填写

1. 被调查者的联系方式（**自愿**留名，以便发现被调查者处于亚健康状态时我们可**反馈**有关信息）

□被调查者签名： □ 联系电话 ：

□调查员签名 ： □ 填表时间 ：

**公务员与健康相关生存质量状况调查问卷**

**亲爱的朋友：**

您好！本次调查由国家教育部人文社会科学研究青年基金项目（编号**10YJCZH192**）和广东省科技计划项目（编号2009B080701073）课题组组织开展，旨在了解公务员与健康相关生存质量状况及其主要影响因素，为卫生部门制定有关的卫生政策和健康保健措施，改善和促进公务员人群的健康状况提供科学的参考依据。

您的每个答案都很重要，请根据您的情况如实填写，尽可能**准确地反映您目前的健康状态**。如果您对某个问题不能肯定回答，请选择最接近您真实感受的答案。

本次调查**不记名**。如果您愿意，请在左边的现场质量控制表中留下您的联系方式。非常感谢您的参与和配合！

**填写示例：**1、您的食欲好吗? □很差 □差 □一般 □好 🗹很好

如果近**四周**内您的食欲很好，就在“很好”前的□中打√。

**第一部分 一般情况**

**性别：**□男 □女 **年龄：** 岁 **民族：** **身高**： cm **体重**： kg

**婚姻状况**： □未婚 □已婚 □离婚 □丧偶 □其他

**学历**：□初中及以下 □中专或高中 □大专 □本科 □研究生

**就职情况：** □在职 □退休 □离休

**单位隶属：** □省级行政/事业管理机构 □地市级行政/事业管理机构

□县市级行政/事业管理机构 □乡镇级行政/事业管理机构

**单位性质：** □政治与行政综合管理机构 □财政经济综合管理机构

□财政经济行业管理机构 □科教文体卫管理机构 □其他

**职务级别：** □国家级正（副）职 □省部级正（副）职 □厅局级正（副）职

□县处级正（副）职 □乡科级正（副）职 □巡查员、副巡查员

□调研员、副调研员 □主任、副主任科员 □科员 □办事员

**工作性质：** □综合管理类 □专业技术类 □行政执法类 □其他

**本人月均收入：** □2500以下 □2500- □5000- □7500- □10000—

**家庭人均月收入：**□2500以下 □2500- □5000- □7500- □10000—

**日均工作时间：**□6小时以下 □6小时- □8小时- □10小时- □12小时及以上

**医疗保障**（可多选）**：**□全自费 □公费医疗保险 □社会医疗保险 □农村合作医疗保险 □商业医疗保险

**居住地：** □城市市区 □城市郊区 □乡镇 □农村 □其他

**居住情况：** □与家人同住 □与朋友同住 □独居 □其他

**第二部分 生活方式及经历事件**

**您是否吸烟？** □不吸 □吸，从开始吸烟到现在有 年

如吸烟，现吸烟量为： □0-3根/天 □4-7根/天 □8根- 1包/天 □1包以上/天

**您是否喝酒？** □从不喝 □很少喝 □经常喝（主要喝 □啤酒 □红酒 □白酒 □其他 ）

如经常喝，从开始喝酒到现在有 年，现每周酒量为：□半斤以下 □0.5斤— □1斤— □1.5斤以上

**您经常吃早餐吗？** □从不吃 □偶尔吃 □经常吃 □每天吃

**您每天的睡眠时间有：** □4小时以下 □4小时- □6小时- □8小时- □10小时及以上

**您经常锻炼吗?** □极少或无 □1-4次/月 □5-8次/月 □9-12次/月 □12次以上/月

**您上下班的交通手段是：**□小汽车 □摩托或电动车 □自行车 □公交车 □步行 □其他

**您的工作应酬多吗？** □极少或无 □1-4次/月 □5-8次/月 □9-12次/月 □12次以上/月

**您每天久坐或长时间伏案、操作电脑吗？** □从不这样 □很少这样 □有时这样 □经常这样 □总是这样

**您认为环境污染对您的健康影响有多大？** □极小或无 □较小 □一般 □较大 □极大

**最近一个月是否有确诊的疾病：** □无 □有，主要疾病名称：

**目前该疾病治愈了吗？** □已治愈 □尚未治愈

如果未治愈，您的病情控制良好吗？　　 □控制良好，无相关症状　□控制不好，症状反复

**最近半年内是否经历以下事件：**（可多选）

□退休 □降职或受批评、处分 □下岗 □迁居 □失恋 □离婚 □与家人不和

□亲人病重或离世 □家庭经济出现问题 □丢失贵重财物 □住房紧张 □工作压力大

□对现职工作不满意 □与上下级关系紧张 □与同事邻居不和 □好友病重或离世

□陷入法律纠纷 □发生事故、意外惊吓、自然灾害 □未经历以上事件

**第三部分 健康测量量表（SF-36）**

下面的问题是询问您对自己健康状况的看法、您的感觉如何以及您进行日常活动的能力如何。如果您对某个问题不能肯定回答，请选择最接近您真实感受的答案，并在相应的○内打√。

**1．总体来讲，您的健康状况是：**

○非常好 ○很好 ○好 ○一般 ○差

**2．跟一年前相比，您觉得您现在的健康状况是：**

○比1年前好多了 ○比1年前好一些 ○和1年前差不多 ○比1年前差一些 ○比1年前差多了

**3．以下这些问题都与日常活动有关。您的健康状况是否限制了这些活动？如果有限制，程度如何？**

|  | 有很多限制 | 有一点限制 | 根本没限制 |
| --- | --- | --- | --- |
| (1)重体力活动（如跑步、举重物、激烈运动等） | ○ | ○ | ○ |
| (2)适度活动（如移桌子、扫地、做操等） | ○ | ○ | ○ |
| (3)手提日杂用品（如买菜、购物等） | ○ | ○ | ○ |
| (4)上几层楼梯 | ○ | ○ | ○ |
| (5)上一层楼梯 | ○ | ○ | ○ |
| (6)弯腰、曲膝、下蹲 | ○ | ○ | ○ |
| (7)步行1 500 米左右的路程 | ○ | ○ | ○ |
| (8)步行800 米左右的路程 | ○ | ○ | ○ |
| (9)步行约100 米的路程 | ○ | ○ | ○ |
| (10)自己洗澡、穿衣 | ○ | ○ | ○ |

**4．在过去4个星期里，您的工作和日常活动有没有**因为身体健康的原因而出现以下这些问题?

|  | 有 | 没有 |
| --- | --- | --- |
| (1)减少了工作或其他活动的时间 | ○ | ○ |
| (2)本来想要做的事情只能完成一部分 | ○ | ○ |
|  | 有 | 没有 |
| (3)想要做的工作或活动的种类受到限制 | ○ | ○ |
| (4)完成工作或其他活动有困难(比如，需要额外的努力) | ○ | ○ |

**5．在过去4个星期里，您的工作和日常活动有没有因为情绪 (如感到消沉或者忧虑**)而出现以下问题：

|  | 有 | 没有 |
| --- | --- | --- |
| (1)减少了工作或其他活动的时间 | ○ | ○ |
| (2)本来想要做的事情只能完成一部分 | ○ | ○ |
| (3)做工作或其它活动不如平时仔细 | ○ | ○ |

**6．在过去4个星期里，您的身体健康或情绪不好在多大程度上影响了您与家人、朋友、邻居或集体的正常社会交往?**

○根本没有影响 ○很少有影响 ○有中度影响 ○有较大影响 ○有极大影响

**7．在过去4个星期里，您有身体上的疼痛吗?**

○根本没有疼痛 ○有很轻微疼痛 ○有轻微疼痛 ○有中度疼痛 ○有严重疼痛 ○有很严重疼痛

**8．在过去4个星期里，身体上的疼痛影响您的正常工作吗（包括上班工作和家务活动）?**

○根本没有影响 ○有一点影响 ○有中度影响 ○有较大影响 ○有极大影响

**9．以下这些问题有关过去1个月里您的感觉，对每一条问题所说的事情，您的情况是怎么**样的?

| 在过去一个月里持续时间 | 所有的  时间 | 大部分  时间 | 比较多  时间 | 一部分  时间 | 一小部  分时间 | 没有这  种感觉 |
| --- | --- | --- | --- | --- | --- | --- |
| (1)您觉得生活充实吗？ | ○ | ○ | ○ | ○ | ○ | ○ |
| (2)您是一个精神紧张的人吗？ | ○ | ○ | ○ | ○ | ○ | ○ |
| (3)您感到垂头丧气，什么事情都不能使您振作起来吗？ | ○ | ○ | ○ | ○ | ○ | ○ |
| (4)您觉得平静吗？ | ○ | ○ | ○ | ○ | ○ | ○ |
| (5)您精力充沛吗？ | ○ | ○ | ○ | ○ | ○ | ○ |
| (6)您的情绪低落吗？ | ○ | ○ | ○ | ○ | ○ | ○ |
| (7)您觉得筋疲力尽吗？ | ○ | ○ | ○ | ○ | ○ | ○ |
| (8)您是个快乐的人吗？ | ○ | ○ | ○ | ○ | ○ | ○ |
| (9)您感觉疲劳吗？ | ○ | ○ | ○ | ○ | ○ | ○ |
| (10)您的健康限制了您的社会活动(如走亲访友)吗？ | ○ | ○ | ○ | ○ | ○ | ○ |

**10．请对下面的每一句话，选出最符合您情况的答案。**

|  | 绝对正确 | 大部分正确 | 不能肯定 | 大部分错误 | 绝对错误 |
| --- | --- | --- | --- | --- | --- |
| (1)我好像比别人容易生病 | ○ | ○ | ○ | ○ | ○ |
| (2)我跟我认识的人一样健康 | ○ | ○ | ○ | ○ | ○ |
| (3)我认为我的健康状况在变坏 | ○ | ○ | ○ | ○ | ○ |
| (4)我的健康状况非常好 | ○ | ○ | ○ | ○ | ○ |

**您大概花了**（ ）**分钟来填完这份调查表。**

本次调查到此结束，再次感谢您的合作！ 祝您健康快乐，工作顺利！
