# Supplementary figures and images for: Lifestyle and health-related quality of life: A cross-sectional study among civil servants in China
Source: BMC Public Health. 2012 May 4;12:330. doi: 10.1186/1471-2458-12-330 (PMC3432623; doi:10.1186/1471-2458-12-330)

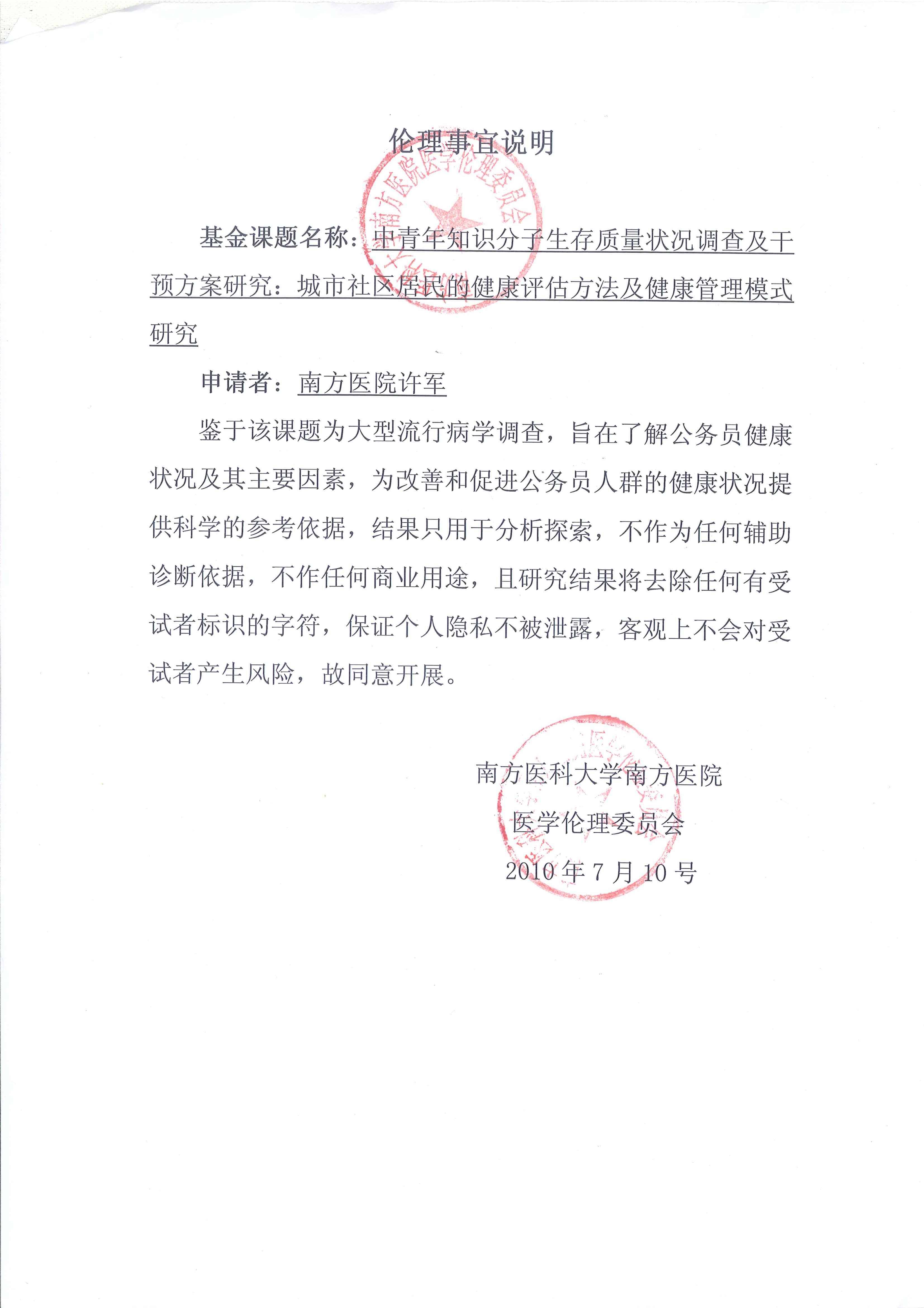

Supplement: Additional file 2 — Questionnaire. [file 1471-2458-12-330-S2.jpeg]
